# Extended in vivo transcriptomes of two ascoviruses with different tissue tropisms reveal alternative mechanisms for enhancing virus reproduction in hemolymph

**DOI:** 10.1038/s41598-021-95553-y

**Published:** 2021-08-12

**Authors:** Heba A. H. Zaghloul, Robert H. Hice, Peter Arensburger, Dennis K. Bideshi, Brian A. Federici

**Affiliations:** 1 Interdepartmental Graduate Program in Microbiology and Institute for Integrative Genome Biology, Riverside, USA; 2 Department of Entomology, University of California, Riverside, Riverside, CA 92521 USA; 3 Department of Biological Sciences, California State Polytechnic University, Pomona, Pomona, CA 91768 USA; 4 Department of Biological Sciences, California Baptist University, Riverside, CA 92504 USA; 5 Department of Botany and Microbiology, Faculty of Science, Alexandria University, Alexandria, Egypt

**Keywords:** Cytoskeleton, Viral pathogenesis

## Abstract

Ascoviruses are large dsDNA viruses characterized by the extraordinary changes they induce in cellular pathogenesis and architecture whereby after nuclear lysis and extensive hypertrophy, each cell is cleaved into numerous vesicles for virion reproduction. However, the level of viral replication and transcription in vesicles compared to other host tissues remains uncertain. Therefore, we applied RNA-Sequencing to compare the temporal transcriptome of Spodoptera frugiperda ascovirus (SfAV) and Trichoplusia ni ascovirus (TnAV) at 7, 14, and 21 days post-infection (dpi). We found most transcription occurred in viral vesicles, not in initial tissues infected, a remarkably novel reproduction mechanism compared to all other viruses and most other intracellular pathogens. Specifically, the highest level of viral gene expression occurred in hemolymph, for TnAV at 7 dpi, and SfAV at 14 dpi. Moreover, we found that host immune genes were partially down-regulated in hemolymph, where most viral replication occurred in highly dense accumulations of vesicles.

## Introduction

Ascoviruses compose a relatively new family of large, enveloped dsDNA viruses (Family *Ascoviridae*) discovered in the late 1970s. They cause chronic diseases, often lasting for weeks before death, in lepidopteran larvae, primarily in species of the family Noctuidae, especially those belonging to the genera *Spodoptera*, *Heliothis*, and *Trichoplusia*. The structure and biochemical composition of their virions, transmission, and pathology at the tissue and cellular level were initially characterized during the 1980s^1–4^. Their virions are large (100 × 400 nm), bacilliform to reniform with a highly reticulate outer membrane surrounding an internal particle consisting of a second membrane that envelops the protein/dsDNA genome. Genomes typically vary from 157 kbp for Spodoptera frugiperda ascovirus 1a (hereafter SfAV), to as much as 190 kbp for Trichoplusia ni ascovirus 2a (hereafter TnAV) and Heliothis virescens ascovirus 3a^4^. SfAV and TnAV belong to the ascovirus species *Spodoptera frugiperda ascovirus 1a* and *Trichoplusia ni ascovirus 2a*, respectively. Phylogenetically, ascoviruses are nucleocytoplasmic large DNA viruses (NCLDVs), which include viruses such as poxviruses, iridoviruses, phycodnaviruses, and mimiviruses that interact with the nucleus but have genes enabling most viral replication to occur in cytoplasmic viroplasms^5–13^. Among the above NCLDVs, based on phylogenetic analyses of major capsid proteins among others, ascoviruses evolved from lepidopteran iridoviruses^14^.

**Figure 1:**
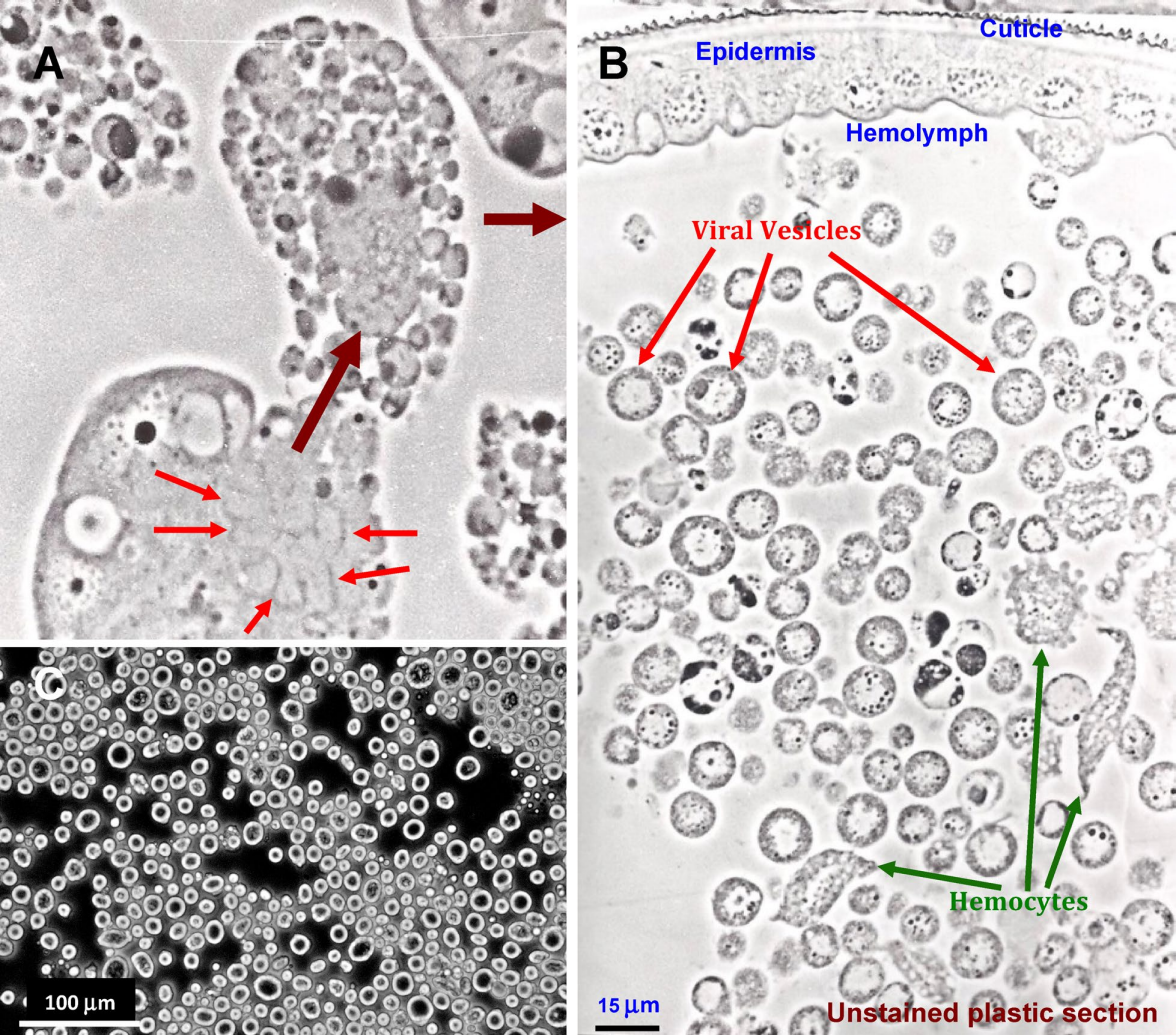
Full cleavage of host cells to form viral vesicles in larvae of *Trichoplusia ni* infected with TnAV^4^. (**A**) Virus-directed cytopathology showing full cleavage of a fat body cell to form viral vesicles. The cell shown is greatly hypertrophied, with the small red arrows pointing to arrays of mitochondria that participate in the de novo formation of membranes that delimit nascent viral vesicles. The lower large maroon arrow points to nascent viral vesicles formed before the basement membrane ruptures, after which they spill into the hemolymph, indicated by the upper maroon arrow. (**B**) Viral vesicles circulating in the hemolymph where most transcription and progeny virion reproduction occurs. The vesicles with dense aggregates along the periphery of the vesicle membrane often have very few virions, and are thought to be spent vesicles, which have released most progeny virions into the hemolymph. (**C**) Phase contrast micrograph of a wet mount of vesicles in undiluted hemolymph of a larvae infected with TnAV^4^.

The structural and biochemical properties of ascovirus virions easily distinguish these viruses from all others. However, their most unique features are their transmission, and to an even greater extent, their cell biology. Typical insect viruses like baculoviruses and cytoplasmic polyhedrosis viruses, are highly infectious by feeding, but ascoviruses are not. Instead, they are easily transmitted mechanically on the ovipositor of parasitoid wasps during oviposition^14–16^. Once a female inserts her ovipositor into an infected larva it becomes contaminated with virions, and subsequent attempts to lay eggs in healthy larvae result in infection. After infecting a cell, replication begins in the nucleus, and within a few hours the nucleus lyses in a process resembling apoptosis, after which the nucleoplasm and cytoplasm intermix^17,18^. Instead of dying, however, the cell hypertrophies markedly, ten-fold or more, and undergoes striking changes in cytoskeletal structure. These include proliferation and movement of mitochondria to cleavage planes within the cell where massive amounts of membrane are synthesized de novo^1,4^. Subsequently, as the first progeny virions form, the enlarged cell is cleaved into numerous viral vesicles. Within two days of infection, the basement membrane of infected tissues begins to weaken and rupture near cells being cleaved, and nascent viral vesicles, typically 3–5 μm in diameter, begin to drift through these ruptures into the hemolymph, circulating there for weeks (Fig. 1)^4^.

Little is known about the molecular genetic strategy ascoviruses use to change cell architecture, generate viral vesicles, and synthesize progeny virions. Nevertheless, the differences between the sites of replication between ascoviruses and other viruses are obvious. As noted above, NCLDVs may begin replication in the nucleus, but most replication, transcription, and virion assembly takes place in the cytoplasm without nuclear lysis. Other viruses typically replicate in either the nucleus or cytoplasm. For example, most RNA viruses of plants and animals replicate exclusively in the cytoplasm^19^, whereas replication of DNA viruses such as adenoviruses, baculoviruses, herpesviruses, and parvoviruses occurs in the nucleus^20^ without nuclear lysis until cell death.

Previous studies of developing ascovirus viroplasms after nuclear lysis demonstrated very few progeny virions were formed prior to viral vesicle formation^1,21^, suggesting most are generated in these vesicles. However, molecular data supporting this remains lacking. In a previous transcriptome study we reported gene expression for the SfAV from 6 h to 7 dpi^22^, focusing on 44 core genes common to ascoviruses, and other genes with transmembrane domains putatively involved in vesicle membrane formation and structure. That study revealed three temporal expression classes, early, late, and very late, and showed genes involved in inhibition of apoptosis were expressed early, followed by very late expression of its caspase, which lyses the nucleus and cleaves most host DNA^17^. Thus, in the present study, we used RNA-Sequencing technology to analyze the transcriptomes of two ascovirus isolates, SfAV and TnAV, over three weeks, choosing these because they differ in tissue tropism. SfAV primarily infects the fat body, which it almost completely destroys within 10 days. Alternatively, TnAV has a broader tissue tropism, infecting the fat body, epidermis, and tracheal matrix, referred to here as somatic tissues, but only infects small areas of each. Here we show that by 7 dpi TnAV transcription has moved from tissues infected initially to the hemolymph, whereas in SfAV this occurs during the second week. Of particular importance are the extremely high expression levels of the major capsid proteins and certain other core proteins that occurred for both viruses in hemolymph, levels much higher than those in the initial tissues infected. These results demonstrate that most virion synthesis occurs in the viral vesicles circulating in the hemolymph, where progeny virions can be acquired for transmission by parasitic wasps during oviposition.

## Results

### Changes in cell architecture directed by ascoviruses

**Figure 2:**
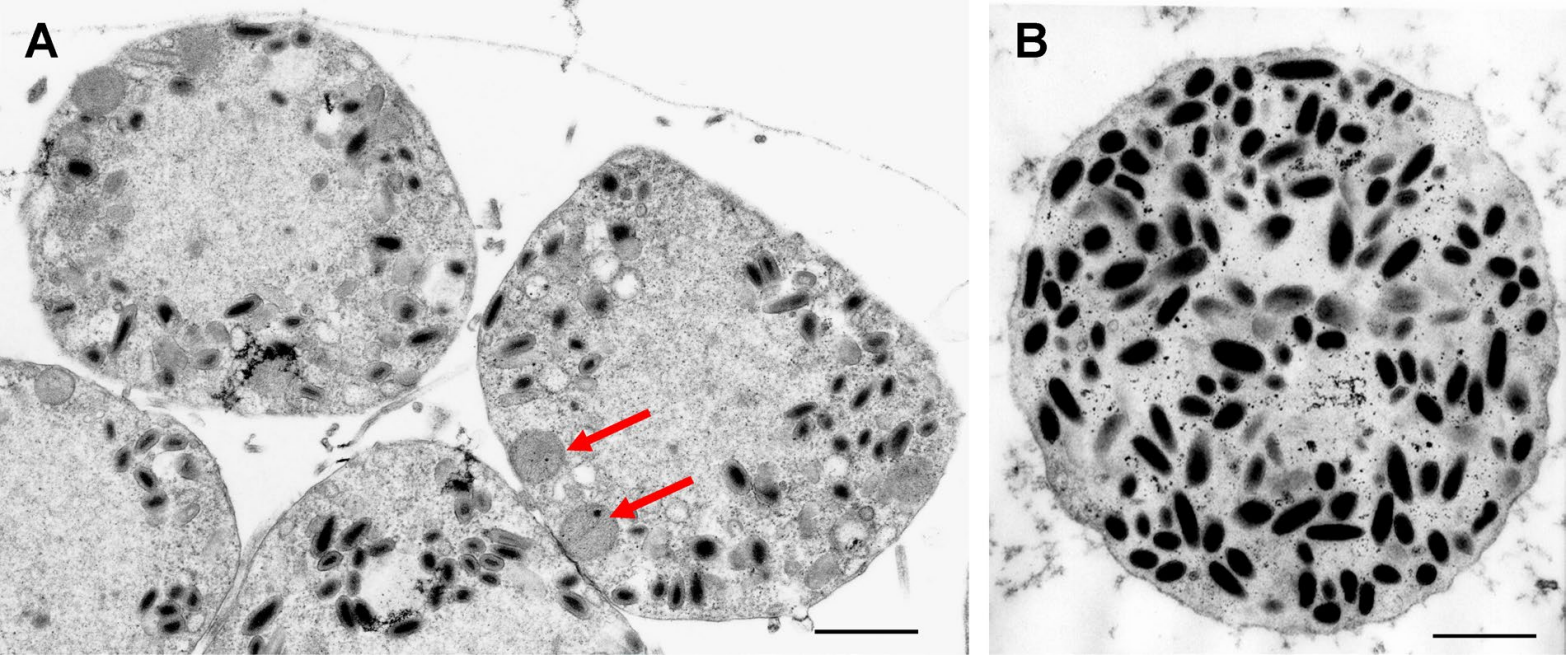
Electron micrographs of nascent and mature vesicles of TnAV. (**A**) Nascent vesicles cleaved from a fat body cell in a *Trichoplusia ni* larva. The thin gray line bordering the top two vesicles is a section through the fat body basement membrane that has not yet ruptured, which will release the vesicles into the hemolymph. Many of the virions at this stage are not completely formed. The red arrows point to mitochondria that participated in the de novo synthesis of the delimiting membrane of the nascent vesicles. (**B**) A grazing section through the periphery of a mature vesicle circulating in the hemolymph. Most virions are now completely formed. Bar in (**A**) and (**B**) represents 800 nm.

The remarkably unique changes in cell architecture directed by ascoviruses summarized in Fig. 1 lead to nascent vesicles that spill into the hemolymph where progeny virions continue to be produced as the vesicles circulate and grow (Fig. 2).

### Virus and host RNA-sequencing mapped read statistics

**Figure 3:**
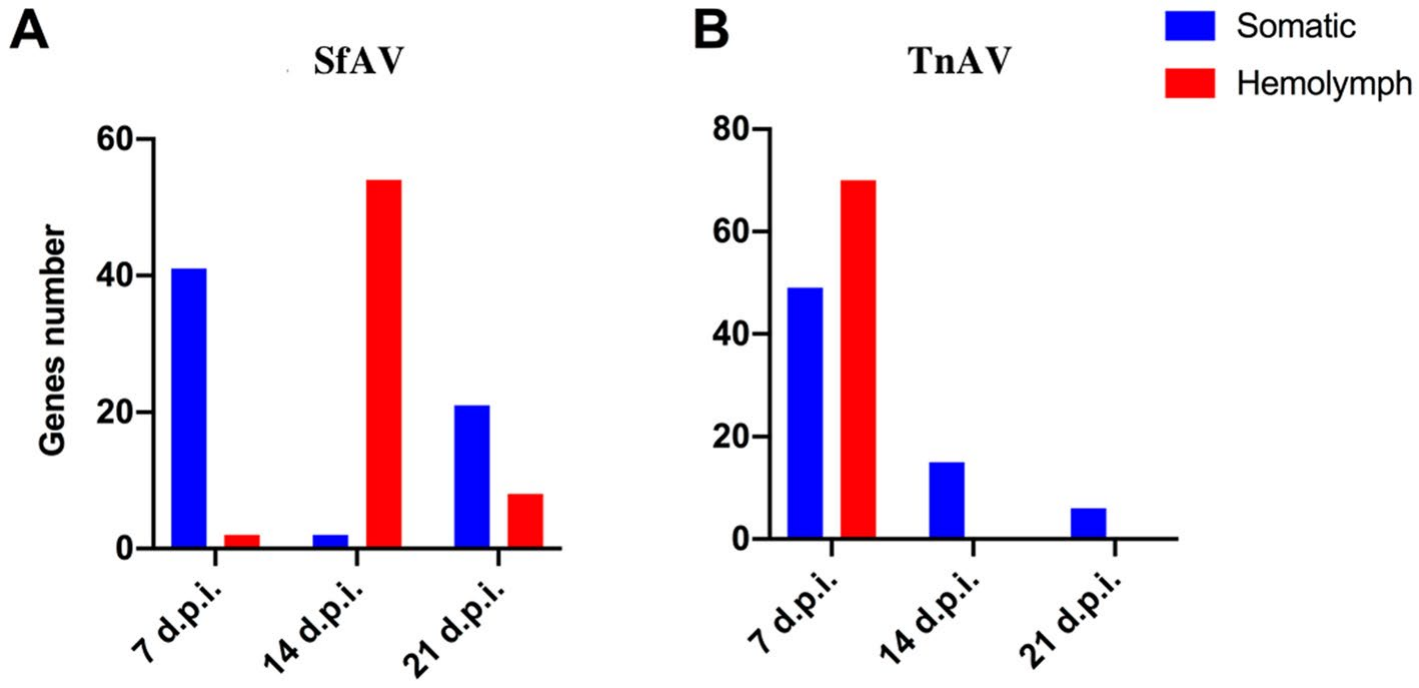
Histogram of the number of key SfAV (**A**) and TnAV (**B**) genes that peak or reach the maximal level of expression in somatic (blue) or hemolymph (red) at 7, 14 and 21 days post-infection. Note that the number of TnAV genes expressed in hemolymph was maximal during the first week post-infection, whereas SfAV genes peaked during the second week.

Comparison of total reads mapped to the virus or host genome demonstrated that in all cases the percent hemolymph virus reads was higher than that of body tissues at all times sampled, specifically, 7, 14, and 21 dpi. For SfAV, the mean percent virus reads for *S. frugiperda* hemolymph was 4.17, 6.45 and 2.34, whereas for the fat body it was 2.19, 1.50 and 2.05 at 7, 14 and 21 dpi, respectively. For TnAV, the mean percent hemolymph virus reads was 29.06, 10.92 and 4.95, while for somatic tissues it was 5.81, 1.92 and 0.35, respectively, for the above sampling times. These results support hemolymph as the main site of ascovirus replication and transcription for both ascovirus isolates despite their difference in tissue tropism. Moreover, for both viruses, transcription of viral genes in the initial tissues infected was maximal by 7 dpi. Subsequently, further supporting the hemolymph as the main site of replication and transcription, the highest level of viral gene expression in this tissue occurred at 7 dpi for TnAV, and 14 days for SfAV (Fig. 3).

### Expression patterns for SfAV and TnAV genes in hemolymph versus somatic tissues

The primary diagnostic signs of ascovirus disease are the opaque whiteness of the hemolymph and prolonged larval life due to retarded development, the latter apparently the result of decreased feeding^16^. For example, the typical time for noctuid larvae from egg to pupation is 10–14 days, whereas larvae infected with ascoviruses survive from three to as many as 6 weeks before dying. Ascovirus vesicles usually begin to appear in the hemolymph two days after infection, and continue to circulate in the blood until larval death. In a previous study of SfAV, we reported the viral transcription pattern during the first 48 h of infection, when replication and transcription were primarily limited to the fat body^22^. Because viral vesicles continue to be produced and circulate in the hemolymph in many larvae for weeks, in the present study we used RNA-Sequencing to study viral gene expression in tissues initially infected by SfAV or TnAV as well as in the viral vesicles circulating in the hemolymph at 7, 14, and 21 days after infection.

Analysis of the SfAV and TnAV core and Trans-Membrane Domain (TMD) genes revealed possible expression clustering into three levels of expression based on the RPKM log_2_ values: (1) low (< 1 to 4), (2) medium (4 to 7) and (3) high (> 7). For SfAV expression of viral genes in somatic tissues, i. e., primarily fat body, was medium at 7, 14, and 21 dpi (Supplementary Fig. S1). Viral gene expression in the hemolymph was either medium or high, depending on the sample date, with, for example, it being high on day 14, when 35 genes were highly expressed. Gene expression at the medium level only exceeded the other two classes on day 21.

The expression of TnAV genes in somatic tissues was low at all sampling dates, i.e., 7, 14, and 21 dpi (Supplementary Fig. S1). However, viral gene expression in the hemolymph was medium to high depending on the sampling date. For example, expression of core and TMD genes was high on day 7 (56 genes) and day 14 (38 genes). Subsequently, not until day 21 was the number of genes (40) expressed at the medium level more than those expressed in the low and high levels.

### Highly expressed viral genes (> 1000 RPKM) at different time points

Transcriptome analysis of SfAV infection in its host revealed that several viral genes were very highly expressed (> 1000 RPKM) at one, two, or all tested time points (Supplementary Fig. S1, and Supplementary Dataset S1A). For SfAV hemolymph, these genes were the lipid membrane protein (ORF035), major capsid protein (ORF041), hypothetical proteins (ORF043, ORF060), yabby-like transcription factor (ORF091**)** and ring finger domain containing protein (ORF097). Two of these ORFs are structural viral genes namely, Major Capsid Protein, MCP (ORF041) and yabby-like transcription factor (ORF091) (Table 1). The remaining four genes (ORF035, ORF043, ORF060, ORF097) are all TMD-containing proteins (Table 2). Five SfAV genes were also highly expressed (> 1000 RPKM) in the fat body, specifically, hypothetical proteins (ORF032, ORF060**)**, BRO-like protein (ORF079), yabby-like transcription factor (ORF091) and ring finger domain containing protein (ORF097). The yabby-like transcription factor (ORF091) is a structural protein, whereas the other ORFs (ORF032, ORF060, ORF097) are all TMD-containing proteins.

**Table 1:** Temporal expression of SfAV and TnAV genes coding for abundant virion structural proteins and enzymes in tissues of their respective hosts, larvae of *Spodoptera frugiperda* and *Trichoplusia ni,* at 7, 14, and 21 days post-infection.

SfAV genes for structural proteins and enzymes	Somatic			Hemolymph		
	7	14	21	7	14	21
ORF009 (DEAD-like helicase of the SNF2 family)	223.28	132.15	115.88	429.58	546.50	167.04
ORF041 (Major capsid protein)	560.29	593.22	785.83	986.79	1708.50	826.11
ORF048 (DNA condensation, P64)	94.95	74.56	86.84	155.62	278.25	93.05
ORF061 (Ervl/Alr family protein)	9.97	19.94	29.28	12.41	36.49	23.66
ORF064 (Serine/threonine protein kinase)	53.70	78.87	113.49	42.29	139.32	109.23
ORF075 (S1/P1 nuclease)	472.73	260.86	194.99	733.32	815.64	349.90
ORF084 (Dynein-like β chain)	12.73	20.23	17.58	13.13	43.85	26.89
ORF091 (High-mobility-group protein/yabby-like protein)	2060.79	2708.71	5202.51	6964.89	14,836.89	5624.16
ORF109 (Haloacid dehalogenase-like hydrolase/CTD-like phosphatase)	140.94	97.69	105.72	223.98	332.07	150.18
**TnAV genes for structural proteins and enzymes**^**a**^						
ORF161 (DEAD-like helicase/SWI/SNF2 family helicase)	3.28	3.62	1.27	175.55	64.06	26.46
ORF153 (Major capsid protein)	185.44	177.17	70.55	10,696.28	4668.94	1525.08
ORF118 (Erv1/Alr family protein)	5.77	5.43	3.11	284.63	139.22	54.05
ORF114_115 (serine/threonine protein kinase)^b^	5.27	4.99	1.84	360.55	144.34	54.78
ORF135 (S1/P1 nuclease)	14.83	13.91	3.38	610.31	211.78	86.93
ORF043 (Dynein-like beta chain)	7.99	8.35	3.32	355.03	148.46	65.31
ORF059 (Yabby-like transcription factor/HMG box)	50.75	36.21	18.22	2185.46	1156.16	413.54
ORF093 (Haloacid dehalogenase-like hydrolase/CTD phosphatase-like protein)	1.33	2.88	2.21	80.76	30.29	18.46
ORF147 (chromosome segregation protein SMC)	20.57	23.00	8.01	1474.73	455.94	133.60

Data presented are the average of three replicates of the Reads Per Kilobase Per Million (RPKM) at each time point for somatic tissues versus hemolymph.

The primary tissue initially infected by SfAV in *S. frugiperda* is the fat body, whereas in *T. ni* the initial tissues infected by TnAV are the fat body, epidermis, and tracheal matrix.

^a^The TnAV-6a (TnAV-2c, previously)^23^ was used as a reference genome to refer to the ORFs order in our TnAV-6a1 variant.

^b^The _ sign refers to two ORFs that were found in our TnAV-6a1 variant strain as a single ORF.

**Table 2:** Temporal expression of SfAV and TnAV genes coding for transmembrane domain containing proteins in tissues of their respective hosts, larvae of *Spodoptera frugiperda* and *Trichoplusia ni,* at 7, 14, and 21 days post-infection.

SfAV-1a genes with predicted transmembrane helix	Somatic			Hemolymph		
	7	14	21	7	14	21
ORF009 (DEAD-like helicase of the SNF2 family)	223.28	132.15	115.88	429.58	546.50	167.04
ORF035 (Lipid membrane protein)	620.83	447.44	378.36	1325.47	1792.00	600.44
ORF054 (Putative myristylated membrane protein)	132.27	95.43	118.61	157.20	252.66	139.14
ORF013 (Esterase/lipase)	67.31	45.96	33.57	31.04	47.27	50.03
ORF087 (Fatty acid elongase)	44.64	29.89	34.86	86.99	87.20	43.46
ORF093 (Patatin-like phospholipase)	20.96	23.48	32.81	51.90	74.58	43.24
ORF112 (Phosphate acyltransferase)	43.51	36.69	54.86	44.38	98.43	62.98
ORF110 (ATPase)	24.72	18.40	16.55	46.20	72.26	28.39
ORF086 (Uvr/REP helicase)	68.83	46.31	51.60	121.24	215.38	106.50
ORF014 (Zinc-dependent metalloproteinase)	30.95	26.64	18.79	44.65	103.21	41.87
ORF097 (RING finger domain)	1169.17	1221.30	1403.31	1959.94	2838.71	2073.95
ORF032 (Hypothetical protein)	1807.96	1300.95	1133.74	164.37	230.75	315.65
ORF062 (Hypothetical protein)	5.45	2.81	3.43	10.89	15.37	7.12
ORF108 (Hypothetical protein)	123.17	96.86	117.62	260.45	360.91	157.95
ORF119 (Hypothetical protein)	95.40	63.67	44.00	212.38	310.68	84.15
ORF007 (Hypothetical protein)	198.77	138.69	85.62	186.47	231.38	280.02
ORF012 (Hypothetical protein)	28.70	25.58	25.27	71.04	133.48	43.09
ORF039 (Hypothetical protein)	53.94	48.48	65.73	110.85	156.91	77.62
ORF045 (Hypothetical protein)	0.21	0.27	0.30	0.10	0.54	0.39
ORF056 (Hypothetical protein)	14.12	15.70	26.43	23.10	45.10	21.94
ORF085 (Hypothetical protein)	206.41	243.02	222.01	263.95	900.86	391.74
ORF092 (Hypothetical protein)	28.31	52.60	74.71	42.92	209.84	102.88
ORF122 (Hypothetical protein)	11.94	8.43	5.66	7.76	17.30	8.94
ORF002 (Hypothetical protein)	75.36	46.69	42.18	203.58	260.88	85.29
ORF043 (Hypothetical protein)	747.85	582.75	884.34	792.14	1388.86	675.05
ORF060 (Hypothetical protein)	745.33	577.04	1456.56	1033.36	1859.18	887.81
**TnAV-6a1 genes with predicted transmembrane helix**^**a**^						
ORF102 (Cathepsin B)	1.29	1.31	0.52	73.41	38.56	13.16
ORF158 (Zinc metalloproteinase)	2.45	2.43	1.60	54.17	36.92	13.83
ORF098 (Phosphate acyltransferase)	3.81	3.46	1.15	219.29	98.14	30.06
ORF46_47 (Fatty acid elongase/elongation of very long chain fatty acids protein 1-like)^b^	37.89	32.49	7.27	1915.30	667.47	228.81
ORF067 (Patatin-like phospholipase)	40.55	33.51	10.50	2645.55	1031.08	290.99
ORF080 (Transcription repressor MOT2/ ubiquitin ligase)	19.80	12.05	5.53	523.56	235.73	78.46
ORF129 (Hypothetical protein/Myristylated membrane-like protein)	11.19	12.97	3.64	1080.61	368.79	100.86
ORF091 (Hypothetical protein)	2.97	2.65	1.61	77.50	32.83	10.12
ORF149 (Hypothetical protein)	340.68	313.75	114.74	20,771.67	7198.21	2683.23
ORF157 (Hypothetical protein)	2.13	2.86	0.96	154.05	80.24	44.71
ORF160 (Hypothetical protein)	1460.48	592.36	110.63	275.46	50.88	57.48
ORF023 (Hypothetical protein)	1666.32	545.18	357.25	2066.77	677.67	378.65
ORF060 (Hypothetical protein)	7.86	4.77	2.56	168.87	75.94	28.18

The prediction of transmembrane helices in proteins was carried out by TMHMM Server v. 2.0. http://www.cbs.dtu.dk/services/TMHMM-2.0/.

Data presented are the average of three replicates of the Reads Per Kilobase Per Million (RPKM) at each time point for somatic tissues versus hemolymph.

The primary tissue initially infected by SfAV-1a in *S. frugiperda* is the fat body, whereas in *T. ni* the initial tissues infected by TnAV are the fat body, epidermis, and tracheal matrix.

^a^The TnAV-6a (TnAV-2c, previously)^23^ was used as a reference genome to refer to the ORFs order in our TnAV-6a1 variant.

^b^The _ sign refers to two ORFs that were found in our TnAV-6a1 variant strain as a single ORF.

The TnAV transcriptome revealed that many of its genes were expressed > 1000 RPKM in hemolymph at one, two, or all tested time points (Supplementary Fig. S1, and Supplementary Dataset S1B). This included 22 ORFs, with the following known or putative functions, three structural genes, MCP (ORF153), yabby-like transcription factor (ORF059) and chromosome segregation protein (ORF147); several lipid metabolism genes, a patatin-like phospholipase (ORF067), a fatty acid elongase (ORF046_ORF047) and lipase (ORF132); nucleotide metabolism proteins, including a lipopolysaccharide modifying enzyme (ORF058), a DNA repair exonuclease SbcCD ATPase (ORF077), a serine/threonine protein kinase (ORF088), a DNA-directed RNA polymerase II (ORF138), and finally an aegerolysin-like protein (ORF029). The others were eleven hypothetical proteins (ORF010, ORF023, ORF052, ORF113, ORF122, ORF129, ORF148, ORF149, ORF150, ORF156, and ORF164) for which the functions are not known. In contrast to these results for hemolymph, the only genes highly expressed in infected *T. ni* somatic tissues were three hypothetical proteins (ORF023, ORF159, ORF160) and an aegerolysin-like protein (ORF029).

### Transcriptome of lepidopteran innate immunity genes in larvae infected with ascoviruses

**Figure 4:**
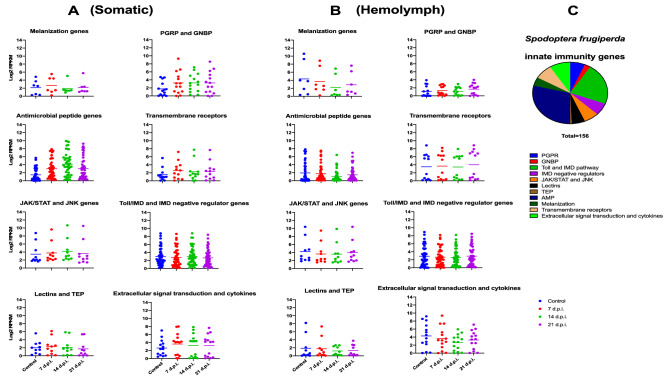
Dot plots representing the expression level of *Spodoptera frugiperda* innate immunity genes post-infection with SfAV in (**A**) somatic tissues (SfAV primarily infects the fat body) and (**B**) hemolymph tissue. (**C**) A pie chart of *S. frugiperda* innate immunity genes included in this study. Each dot refers to an innate immunity gene in the specified pathway. Horizontal lines show the mean value.

The transcriptome of 156 annotated immunity-associated genes identified previously in the *S. frugiperda* genome^24^ was analyzed in the fat body and hemolymph. These genes included representatives for Gram-negative bacteria binding-protein (GNBP), peptidoglycan recognition protein (PGRP), lectins, thioester containing protein (TEP), transmembrane receptors, extracellular signal transduction and cytokines, intracellular signaling pathways (for example: toll pathway, imd pathway, negative regulators of imd pathway, JAK/STAT and JNK pathways) and effectors (for example: phenoloxidase system, antimicrobial peptides, lysozymes and lysozyme-like proteins and others; Supplementary Dataset S1C,D). Dot plot representation of the expression of these genes demonstrated SfAV provoked an immune response in *S. frugiperda* larvae in different tissues (Fig. 4). However, in hemolymph, where the vesicles dominate and virus replication peaked at 14 dpi (Fig. 3 and Supplementary Fig. S1), showed a simultaneous reduction in the immune response, especially when compared to 7 and 21 dpi. Specifically, 60 genes reached the minimum value of their expression over the three sampling times. In other tissues, most of the innate immunity genes showed the maximum value of their expression at 14 dpi. Specifically, 75 genes were expressed by their peak value at this time (Fig. 4). The unpaired t-test of *S. frugiperda* innate immunity genes log_2_ RPKM values at 14 dpi revealed a very highly significant difference between somatic and hemolymph tissues (p value is less than 0.0001). This result implies that the immune system of *S. frugiperda* encounters a spatial and temporal partial silencing during this active virus replication stage in the hemolymph.

**Figure 5:**
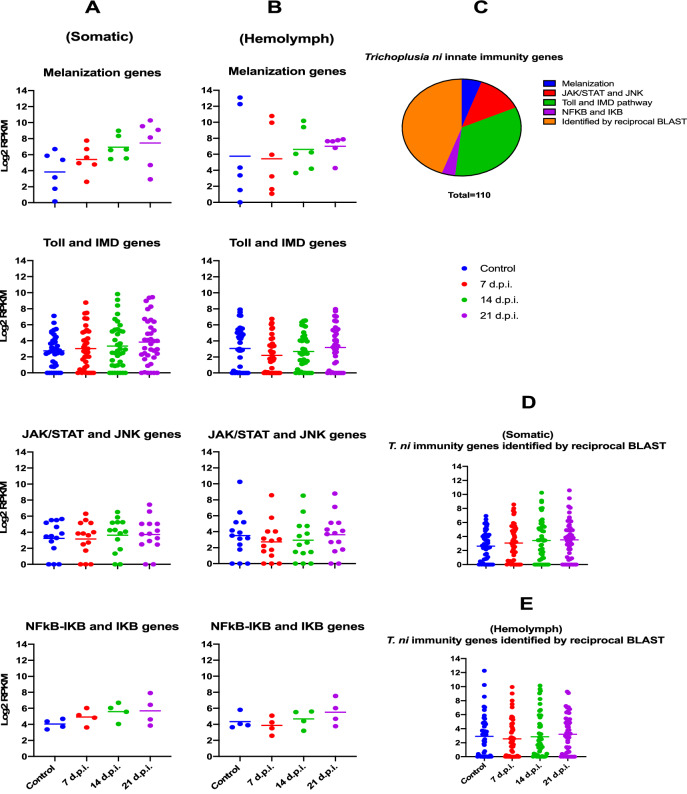
Dot plots representing the expression level of *Trichoplusia ni* innate immunity genes post-infection with TnAV. (**A**) somatic tissues (TnAV initially infects somatic tissues such as fat body, epidermis and tracheal matrix) and subsequently, (**B**) hemolymph tissue. (**C**) A pie chart of *T. ni* innate immunity genes included in this study. Dot plots in (**D**) and (**E**) display the expression levels of innate immunity genes identified by reciprocal BLAST in *T. ni* somatic and hemolymph tissues, respectively. Each dot refers to an innate immunity gene in the specified pathway. Horizontal lines show the mean value .

Similarly, dot plot representation of innate immunity gene expression illustrated TnAV provoked an immune response in different *T. ni* tissues (Fig. 5). Interestingly, the transcriptome of *T. ni* immunity-associated genes identified previously^25^ or in our study using reciprocal blast searches (Supplementary Dataset S1D,F–H) post TnAV infection were similar to *S. frugiperda* immunity genes in blood tissue. For example, TnAV genes expressed at day 7 post-infection, in all tissues, as assessed by their peak RPKM values, were higher compared to the same genes at 14 and 21 dpi (Fig. 3). As in SfAV infection, innate immunity gene expression was down regulated during this active stage of viral replication or transcription (Fig. 5). However, the *T. ni* response differed from *S. frugiperda* in that expression of innate immunity genes decreased not only in hemolymph, but also in the somatic tissues during this virus active replication stage. Nevertheless, we noticed the decreased expression of *T. ni* innate immunity genes in hemolymph was higher than in somatic tissues at day 7 post-infection. The unpaired t test of *T. ni* innate immunity genes at 7 dpi revealed that the difference is not quite statistically significant (p value equals 0.0833). Overall, this makes the silencing of innate immunity genes a unique response to ascovirus infection, especially in the hemolymph, where dense accumulations (Fig. 1) of vesicles active in virus replication occur.

## Discussion

Ascoviruses, like other DNA viruses, initiate replication in the nucleus. However, it remains unknown how replication continues after their caspase and similar proteins cause nuclear lysis^17,18^. Since ascoviruses cause a chronic disease that lasts for weeks, we expanded our previous in vivo transcriptomic studies^22,26^ by extending our SfAV experiments to three weeks and making these comparative by including TnAV. We compared hemolymph transcriptomes at 7, 14, and 21 days for both viruses with those of the other primary body tissues at the same time points. As noted above, SfAV infection is limited primarily to the fat body, which is almost totally destroyed over a period of 10–14 days, whereas TnAV infects multiple small regions of most body tissues, especially major tissues such as the fat body, epidermis, and tracheal matrix^3,16,21^. The results of these extended comparative experiments demonstrated both viruses expressed genes in the hemolymph, i.e., viral vesicles, as well as the somatic tissues at all time points sampled (Supplementary Fig. S1). There were differences in the two viruses in that TnAV hemolymph and somatic tissue reads where highest at 7 dpi and then declined by 14 and 21 days, whereas SfAV reads for both fluctuated, possibly a result from the fat body being the only major body tissue infected other than hemolymph. In any case, these results make it clear transcription occurred in the viral vesicles circulating in the hemolymph during the first three weeks of infection. Thus, more than any other insect virus, ascoviruses take advantage of their hosts by producing progeny virions in the hemolymph, a tissue rich in nutrients including amino acids, carbohydrates such as glucose and trehalose, numerous proteins and lipids, and inorganic salts^27^.

Our experiments also revealed an interesting apparent difference in infection and replication strategies between SfAV and TnAV, which we attribute to their different tissue tropisms. Both viruses had similar numbers of genes expressed, ascertained by their maximum values in the tested time points in somatic tissues during the first week of infection, which then declined in the second and third weeks (Fig. 3). However, in hemolymph the largest number of TnAV genes expressed as determined by their maximum values occurred during the first week, whereas for SfAV the largest number occurred during the second week. Therefore, TnAV’s replication program appears to first infect only limited regions of the primary somatic tissues, i.e., fat body, epidermis and tracheal matrix, after which the nascent vesicles spill quickly into the hemolymph, possibly accelerating virion synthesis and transmission. In SfAV, the largest number of viral genes peaked in expression during the second week, a strategy that correlates with maximizing formation of nascent viral vesicles within the fat body during the period when this tissues basement membrane undergoes significant deterioration and rupturing throughout this tissue, enabling large numbers of nascent vesicles to enter the hemolymph^3,16,21^.

Major support for the important role viral vesicles play in ascovirus reproduction is demonstrated by the highly expressed viral genes (> 1000 RPKM) at late stages of SfAV and TnAV infection in their hosts. For example, the genes for MCP and other structural proteins were highly expressed in hemolymph for both viruses (Table 1), implying the importance of these proteins for synthesizing new virions. Moreover, the increase in some SfAV Transmembrane-Domain containing (TMD) genes in hemolymph and fat body, suggests these proteins play a role in the structure of the outer vesicle membrane and immune system evasion (Table 2).

Another interesting result is that the TnAV aegerolysin gene was highly expressed in *T. ni* body tissues and hemolymph, suggesting this protein is important for virus reproduction. Aegerolysins are low in mass (15–17 kDa) with characteristic β-sheet structure, and occur widely in fungi, bacteria, plants, insects and protozoa where their biological roles are diverse, including antibacterial, antitumor, and antiproliferative effects^28,29^. They interact with lipid membranes and vesicles, inducing pore formation and permeabilization. TnAV and Heliothis virescens ascovirus (HvAV) are the only viruses known to encode aegerolysin gene homologs, and thus they may play a role in their broad tissue tropism.

A reported role of viroplasms is to conceal or protect the viral genome and transcripts from the host antiviral defenses^9^. The expression of host innate immunity genes decreased or was silenced at least partially where vesicles were abundant, especially at 7 and 14 dpi, respectively, for both viruses in the hemolymph (Figs. 4 and 5). The time points tested had the maximum expression values for these viral genomes (Fig. 3 and Supplementary Fig. S1). Thus, these results imply an additional role for viral vesicles in host immune system evasion. The mechanisms by which ascoviruses control host gene expression are not known. However, being DNA viruses increases the possibility that ascoviruses use microRNAs (miRNAs) to alter host gene expression. For example, herpesviruses use miRNAs in host immune system evasion, prolonging infected cell longevity and postponing lysis induction^30^. Other DNA viruses are known to encode miRNAs, for example, polyomaviruses, adenoviruses, as well as insect baculoviruses^31^. Ascoviruses use viral miRNAs, as reported in case of Heliothis virescence ascovirus (HvAV-3e), which uses HvAV-miR-1, identified in the gene coding for the Major Capsid Protein, MCP, which down-regulates the viral DNA polymerase inhibiting virus replication^32^. Moreover, ascoviruses possess a conserved RNaseIII endonuclease enzyme that is reported in HvAV-3e to silence the host RNA interference (RNAi) response^33^. In general, more research is needed to identify how ascoviruses can silence or down-regulate host immunity-associated genes on a wide scale to avoid interfering with viral gene replication and expression, as has also been shown for HvAV-3h^34^.

Overall, the ascovirus viral vesicle represents a unique system for virus replication not known previously. One of the most fascinating aspects of this system is how it evolved, which molecular data suggest was from lepidopteran iridoviruses^14^. Iridoviruses are now known to be very common, although fifty years ago they were thought to be restricted to insects, having originally being found in larvae of cranefly and then many other insects^12^. Subsequently, they were reported from crustaceans and vertebrates, especially fish^35^. One of the most interesting facets of iridovirus biology is that typically they are poorly infectious by feeding, and do not generally infect the midgut epithelium (stomach), at least in invertebrates. This is also characteristic of ascoviruses, supporting their evolution from iridoviruses. The mechanisms by which iridoviruses infect their hosts remain unclear, with entry through cuticle injuries being one, via tracheoles another, and by transovarial transmission (which would also require a previous breach of cuticle or tracheal matrix). In any case, in contrast to baculoviruses and cypoviruses, which are transmitted primarily by feeding and are common in certain insects, especially larvae in lepidopteran populations, where the former can cause epizootics, iridovirus infections in invertebrate populations are extremely rare^12^. Thus, though there are few studies of ascovirus prevalence in noctuid populations, they appear to be much more common than baculoviruses, especially in larval populations late in the season when parasitoid populations are high. For example, ascovirus prevalence ranges from 10 to greater than 70% in populations of *Heliothis* and *Helicoverpa* populations feeding on cotton in the southeastern United States^36^.

It is now clear that the differences between the cell biology of iridoviruses and ascoviruses are extraordinary, and that the latter apparently evolved from the former by acquiring numerous genes coding for regulatory and structural genes from their lepidopteran hosts, and possibly some through exchanges with other viruses when they replicate in the same cell, as rare as that might be. Thus, given the diversity of phylogenetic branches of the iridovirus tree, the only one that accumulated enough genes during evolution to greatly increase its prevalence in host populations was the branch that became the ascoviruses. Ascoviruses are so different from iridoviruses that their unique properties justified the establishment of a new virus family, *Ascoviridae*^37^. The reason they were discovered relatively recently is that in field populations of noctuid larvae ascoviruses lack obvious signs of disease, the main sign being retarded development likely due to decreased feeding^16^. Thus, ironically, whereas the prevalence of iridoviruses in lepdiopteran populations is extremely low^12^, it is possible genes acquired by ancestral lepidopteran iridoviruses while replicating in their hosts led to the evolution of ascoviruses, making them one of the most common types of viruses that occur and cause death routinely in noctuid populations, facilitated by female parasitoid wasps breaching the cuticle with ovipositors contaminated with ascovirus virions and vesicles.

The function of many genes in complex DNA viruses remain unknown, which applies to ascoviruses as well, especially because of their recent discovery. Many of the genes evolved from iridovirus genes, such as the major capsid protein, which along with other virion structural proteins likely resulted in changing the virion shape from being icosahedral in iridoviruses to bacilliform or reniform in ascoviruses. While clearly speculative, the increased surface length of ascoviruses (300–400 nm) virions versus iridovirus triangular plates (< 100 nm) likely increased the contact surface between these and female wasps’ ovipositor, facilitating transmission. While not well documented, the hemolymph of larvae infected with ascoviruses is dense with progeny virions, apparently released by viral vesicles and/or liberated as these deteriorate during circulation. Another virion change identified previously is that rather than having small polyamines that neutralize the charges on DNA to encapsidate the genome, ascoviruses use a novel protein, 64 kDa (ORF 048), to neutralize and package the genomic DNA^38,39^.

Finally, another important question to be answered is whether the ascovirus viral vesicle can generate sufficient resources to fully support viral replication? There are two scenarios we can speculate based on this RNA-Sequencing study and previous physiological and biochemical studies^40^. On the one hand, the nucleus and nuclear DNA are not destroyed immediately after the virus entry and hence all replication precursors may be formed during this period (i.e., before nucleus lysis). On the other hand, SfAV infection is limited primarily to the fat body, whereas TnAV infects multiple but small regions of somatic tissues. Thus, for both viruses the source of necessary replication precursors for circulating vesicles may be uninfected regions of somatic tissues. Indeed, studies of ascoviruses are still in the preliminary stages, and thus future studies will likely reveal many novel mechanisms that have evolved to manipulate cell biology, and with relatively few genes compared to the genomes of their eukaryotic hosts. Among others, yet to be elucidated are molecular control of cell hypertrophy and reorganization, proliferation and movement of mitochondria, mechanisms that control massive synthesis of membranes that form the viral vesicles along cleavage planes, and structure and composition of the delimiting membranes of the viral vesicles. Aside from understanding ascovirus biology, identifying the function of the proteins encoded by ascovirus genes may provide insight into how to restore the function of degenerate mitochondria characteristic of many somatic neurological diseases in vertebrates^41^.

## Materials and methods

### Ascovirus infection of lepidopteran hosts

Two ascovirus isolates were included in this comparative study, namely the Spodoptera frugiperda ascovirus 1a (SfAV-1a) and a minor variant of the Trichoplusia ni ascovirus 6a, TnAV-6a1. Hereafter these isolates are referred to as SfAV and TnAV. SfAV has a narrow tissue tropism, replicating primarily in the fat body tissue. It also has a narrow host range, replicating mainly in *Spodoptera* species. This is in contrast to TnAV, which has a broad tissue tropism and host range^4,16^. Based on our previous research^22^, in the current study we conducted a more comparative transcriptomic analysis in different tissues of larvae infected as 3rd instars of *Spodoptera frugiperda* or *Trichoplusia ni*, and for three weeks after infection. SfAV or TnAV were used, respectively, to infect *S. frugiperda* or *T. ni*. To mimic the parasitic wasp infection in nature, a minutin pin was contaminated with virions of SfAV or TnAV and inserted into the 3rd instar larval abdomen. The pin was dipped in a suspension of viral vesicles (10^8^/vesicles/mL). For controls, the pin was dipped into phosphate buffered saline (PBS). During the weeks of study (21 days), all larva were grown at room temperature (22 °C) and supplied periodically with general noctuid artificial diet prepared according to the manufacturer instructions by dissolving 162 g dry mix to 930 mL boiling water for 1 L preparation (Benzon Research, Carlisle, PA).

### Total RNA isolation from host body tissues and hemolymph

Total RNA was extracted with TRIzol (Invitrogen, Life Technologies) from healthy and infected lepidopteran hosts following the manufacturer’s instructions. Total RNA was collected at 7, 14 and 21 days post-infection. For each sample, RNA was isolated from hemolymph or a combination of other body tissues, which from the standpoint of bulk, consisted primarily of the fat body, epidermis, tracheal matrix and midgut. Hemolymph was collected by piercing the cuticle and allowing the blood to drip into Trizol, followed by 30 s of mixing by vortexing. The other body tissues were transferred to Trizol and triturated for efficient homogenization. For healthy or PBS buffer-injected control larvae, RNA was collected at the time of injection (0 h). RNA was harvested from three biological replicates; this was repeated at all subsequent time points. In total 48 samples were collected. The number of *S. frugiperda* and *T. ni* larvae contributed to each biological replicate was 4, 2, 2 and 1 for control, 7, 14 and 21 dpi, respectively. Since our experiment is qualitative, the included infected larvae needed to pass the microscopic examination first. For instance, the infection was confirmed by the presence of white hemolymph and by microscopy to make sure this tissue was packed with viral vesicles prior to RNA collection^22^.

### DNase-treatment, preparation of RNA-sequencing libraries, and sequencing

The collected total RNA samples were cleaned, concentrated and treated with DNAse following the manufacturers’ instructions for, respectively, RNA Clean and Concentrator TM-5 kit (Zymo Research) and RNase-free DNase set (Qiagen). RNA quantity and quality were determined using a Thermo Scientific NanoDrop ND-2000c spectrophotometer. The cDNA libraries were constructed following the protocol of NEBNext Ultra Directional RNA Library Prep Kit (New England Biolabs) for Illumina. Three biological replicates were sequenced for each time point for each tissue included in this study. An Agilent 2100 Bioanalyzer was used to ensure the quality of the pooled libraries before sequencing using the NextSeq500 sequencer (Illumina) in Core Facility at the UCR Institute for Integrative Genome Biology.

### Identification and classification of virus and host genes

The SfAV core and Transmembrane-Domain containing (TMD) genes included in this study are listed in Supplementary Dataset S1. The TnAV genome (GSE114902) used in this study was assembled based on the publicly available TnAV-6a genome in the NCBI database (Accession number: DQ517337.1). Thirty-four contig sequences were generated using Trinity software with “genome guided” assembly option^42^. The TnAV-6a1 core, TMD-containing and species-specific genes are listed in Supplementary Dataset S1B.

The *S. frugiperda* and *T. ni* host genes (Supplementary Dataset S1C–H) were either identified in previous studies^24,25^ or identified in our study using reciprocal blast searches. Specifically, *S. frugiperda* (Corn variant) genes were used as queries to identify orthologous genes in the *T. ni* genome (accession number: ASM360422v1) http://www.tnibase.org/cgi-bin/index.cgi.

### Bioinformatics analysis, libraries statistics and RPKM calculations

Low sequencing quality and adapter sequences were removed from the libraries using trimmomatic-0.32 and ribosomal contaminants were removed with SortMeRNA version 2.0^43^. Sequencing reads were mapped to one or several of the following reference genome assemblies: (1) viral genome of SfAV (described above), (2) viral genome of TnAV (described above), (3) *Spodoptera frugiperda genome,* SF_CORN_1 assembly (NCBI accession GCA_900240015.1), (4) *Trichoplusia ni* genome (NCBI accession ASM360422v1^44^ and bowtie2^45^ and the splice-aware program hisat2^46^ was used for all other genomes. Library statistics, read counts and RPKMs calculation were determined using the bedtools^47^ intersectBed program in conjunction with custom written Perl scripts (available upon request). RPKM values were calculated for each replicate individually using the standard RPKM calculation formula^48^. RPKM values from the three replicates were then averaged to obtain a single estimate of transcript expression. The TnAV-6a (previously TnAV-2c)^23^ was used as a reference genome to refer to the ORFs order in our TnAV-6a1 variant. GraphPad Prism version 8.4.3 (GraphPad Software, San Diego, California, USA, www.graphpad.com) was used for Figs. 3, 4, 5 and Supplementary Fig. S1 generation and unpaired t-test was used to compare the statistical difference of the innate immunity genes expression level between the different tissues.

### Light and electron microscopy

For light and transmission electron microscopy, pieces fat body, epidermal, and tracheal matrix tissue, approximately 2 mm^3^, were dissected from healthy and infected cabbage looper (*T. ni*) and fall armyworm (*S. frugiperda*) larvae at 7 and 14 days post-infection. Hemolymph samples of 300 µl from infected larvae were sedimented to pellet viral vesicles and hemocytes. All tissues were fixed in 3% glutaraldehyde in 2% cacodylate buffer, post-fixed with 1% osmium tetroxide, and embedded in Epon-Araldite blocks. Plastic sections were cut with an ultramicrotome and observed either with phase contrast or electron microscopy (EM). Ultrathin sections were stained with lead citrate and uranyl acetate.

## Supplementary Information

Supplementary Information.

## Data Availability

The data generated in this study has been deposited to the NCBI GEO data repository under series record GSE174236.
