# Supplementary material for: Extended in vivo transcriptomes of two ascoviruses with different tissue tropisms reveal alternative mechanisms for enhancing virus reproduction in hemolymph
Source: Sci Rep. 2021 Aug 12;11:16402. doi: 10.1038/s41598-021-95553-y (PMC8361023; doi:10.1038/s41598-021-95553-y)
Supplement: Supplementary file 1 — Supplementary Information. [file 41598_2021_95553_MOESM1_ESM.docx]

**Supplementary File**

**Extended *in vivo* transcriptomes of two ascoviruses with different tissue tropisms reveal alternative mechanisms for enhancing virus reproduction in hemolymph**

Heba A. H. Zaghloul ^a, e^, Robert H. Hice ^a^, Peter Arensburger ^c^, Dennis K. Bideshi ^d^, and Brian A. Federici ^a, b^

^a^ Interdepartmental Graduate Program in Microbiology and Institute for Integrative Genome Biology.

^b^ Department of Entomology, University of California, Riverside, Riverside, CA 92521.

^c^ California State Polytechnic University, Pomona, Department of Biological Sciences, 3801 West Temple Avenue, Pomona CA 91768.

^d^ Department of Biological Sciences, California Baptist University, Riverside, CA 92504.

^e^ Department of Botany and Microbiology, Faculty of Science, Alexandria University, Egypt.

* corresponding author name: Brian Federici

**Email:** brian.federici@ucr.edu

**This supplementary file includes:**

Figure S1

Legends for Dataset S1(A-L)

S References

**Figure S1**. Heat-map representing temporal expression of SfAV **(A)** and TnAV **(B)** core, transmembrane domain-containing genes, and species-specific genes in their respective hosts, larvae of *Spodoptera frugiperda* and *Trichoplusia ni,* somatic and hemolymph tissues*.* The expression time scale ranged from uninfected control at 0 h to 21 days post-infection. The color scale represents the percentage normalized Reads Per Kilobase Per Million value from an average three replicates taken at each time point (RPKM; i.e., the highest gene RPKM value represents 100% and the lowest value represents 0%). The ORF numbers for TnAV were derived from the order of ORFs in the TnAV genome (Accession number: DQ517337.1). The “_” sign refers to two ORFs that were found in our minor TnAV-6a1 variant genome sequence as a single ORF. The background expression level of all viral genes determined in control samples was subtracted from all the time points tested except in cases of zero values. The gene identifications, their putative function, mean RPKM and RPKM standard deviation values are listed in **Dataset S1A and S1B.** GraphPad Prism version 8.4.3 was used to generate the above figure (GraphPad Software, San Diego, California, USA).

Dataset S1 (separate Excel file).

S1A Spodoptera frugiperda ascovirus-1a core and transmembrane domain containing genes expression in somatic and hemolymph tissue of *Spodoptera frugiperda* larvae.

S1B Trichoplusia ni ascovirus-6a1 core, transmembrane domain containing genes and species specific genes expression in somatic and hemolymph tissue of *Trichoplusia ni* larvae. Open Reading Frames number is derived from the TnAV-6a (TnAV-2c, previously) (1).

S1C *Spodoptera frugiperda* innate immunity genes (2) expression levels in somatic tissues post infection with SfAV-1a.

S1D *Trichoplusia ni* innate immunity genes (3) expression levels in somatic tissues post infection with TnAV-6a1.

S1E *Spodoptera frugiperda* innate immunity genes (2) expression levels in hemolymph tissue post infection with SfAV-1a.

S1F *Trichoplusia ni* innate immunity genes (3) expression levels in hemolymph tissue post infection with TnAV-6a1.

S1G *Trichoplusia ni* innate immunity genes (identified using reciprocal blast searches) expression levels in somatic tissues post infection with TnAV-6a1.

S1H *Trichoplusia ni* innate immunity genes (identified using reciprocal blast searches) expression levels in hemolymph tissue post infection with TnAV-6a1identified by reciprocal BLAST.

S1I *Spodoptera frugiperda* libraries statistics in somatic tissues post infection with SfAV-1a

S1J *Spodoptera frugiperda* libraries statistics in hemolymph tissue post infection with SfAV-1a

S1K *Trichoplusia ni* libraries statistics in somatic tissues post infection with TnAV-6a1

S1L *Trichoplusia ni* libraries statistics in hemolymph tissue post infection with TnAV-6a1

**S References:**

1. L. Wang, J. Xue, C.P. Seaborn, B.M. Arif, X.W. Cheng, Sequence and organization of the *Trichoplusia ni* ascovirus 2c (*Ascoviridae*) genome. *Virol.* **354**, 167–177 (2006).
2. A. Gouin et al., Two genomes of highly polyphagous lepidopteran pests (*Spodoptera frugiperda*, Noctuidae) with different host-plant ranges. *Sci. Rep.* **7**, 11816 (2017).
3. A. Shrestha, K. Bao, W. Chen, P. Wang, Z. Fei, G. W. Blissard, Transcriptional responses of the *Trichoplusia ni*midgut to oral infection by the baculovirus Autographa californica multiple nucleopolyhedrovirus. *J Virol.* **93**:e00353-19 (2019).
